# Use of naloxone by EMS for opioid-associated out-of-hospital cardiac arrest and associated patient-centered outcomes: A systematic review

**DOI:** 10.1371/journal.pone.0351738

**Published:** 2026-06-17

**Authors:** Hania Siddiqui, Khadija Brouillette, Chris Walsh, Sheldon Cheskes, Ian R. Drennan, Charles D.G. Keown-Stoneman, Steve Lin, Rohit Mohindra

**Affiliations:** 1 Institute of Medical Science, University of Toronto, Toronto, Ontario, Canada; 2 Li Ka Shing Knowledge Institute, St. Michael’s Hospital, Unity Health Toronto, Toronto, Ontario, Canada; 3 Faculty of Medicine and Health Sciences, McGill University, Montreal, Ontario, Canada; 4 Library Services, Sinai Health, Toronto, Ontario, Canada; 5 Sunnybrook Centre for Prehospital Medicine, Sunnybrook Health Science Centre, Toronto, Ontario, Canada; 6 Department of Family and Community Medicine, Division of Emergency Medicine, University of Toronto, Toronto, Ontario, Canada; 7 Ornge Critical Care Transport, Mississauga, Ontario, Canada; 8 Dalla Lana School of Public Health, University of Toronto, Toronto, Ontario, Canada; 9 Division of Emergency Medicine, Department of Medicine, University of Toronto, Toronto, Ontario, Canada; 10 North York General Hospital, Toronto, Ontario, Canada; 11 Schwartz Reisman Emergency Medicine Institute, Toronto, Ontario, Canada; UT Health San Antonio: The University of Texas Health Science Center at San Antonio, UNITED STATES OF AMERICA

## Abstract

**Objective:**

As opioid-related fatalities continue to climb, it is imperative to advance our understanding of the management of life-threatening opioid emergencies, including cardiac arrest. Emergency medical services (EMS) administered naloxone reverses critical respiratory depression within minutes; however, the role of naloxone in cases of opioid-associated cardiac arrest (OA-OHCA) is unknown. This systematic review sought to summarize patient outcomes following EMS-administered naloxone in OA-OHCA cases.

**Methods:**

Following PRISMA guidelines, a systematic search was conducted in OVID Medline, Embase, and Cochrane from database inception to December 2024. Original, peer-reviewed studies examining patients with OA-OHCA who were given naloxone by EMS were included. Two independent reviewers screened titles/abstracts and full-texts in Covidence based on predetermined inclusion criteria. Relevant data points were extracted, and a risk of bias assessment was conducted for included studies. No meta-analysis was performed due to heterogeneity across the included studies.

**Results:**

The literature search yielded 4814 articles, of which 8 studies met eligibility and were included. Seven of the included studies were retrospective cohort studies conducted in the United States. The eighth included study was a prospective cohort study conducted in Denmark. The total sample size for drug-related OHCA patients was 1294 (range 16–471) from all the included studies. Risk of bias was assessed to be low to moderate in seven studies and serious in one study. A minority of patients achieved return of spontaneous circulation (ROSC), with ROSC ranging from 4.3% to 50%. Survival to hospital admission ranged from 11.1% to 55%, while survival to hospital discharge ranged from 0% to 20.4%.

**Conclusions:**

There are a limited number of studies assessing the use of naloxone in patients who present in opioid-associated out-of-hospital cardiac arrest (OA-OHCA). Further research is needed to evaluate the effectiveness of naloxone in this patient population.

## Introduction

The opioid epidemic in North America is responsible for the death of thousands of individuals annually [1,2]. Drug overdoses, the majority of which are due to opioids, are the leading cause of death for adults under the age of 50 in the United States [3] and are the leading cause of major accidental death in Canada [4]. Opioid-associated out-of-hospital cardiac arrest (OA-OHCA) is the most severe manifestation of opioid use and is increasingly becoming identified as a significant cause of OHCA [5,6].

Airway obstruction and respiratory failure are responsible for the progression of most opioid overdoses to an OA-OHCA. The mechanism of action is well established, wherein excessive opioid use inhibits the neural activity of respiratory centers in the brainstem while also reducing the carbon dioxide sensitivity of brainstem chemoreceptors [5–7]. This combination leads to respiratory depression, which can progress to respiratory arrest. Additionally, opioids can cause cardiac toxicity by inhibiting sodium and potassium channels critical for maintaining the heart’s pacemaker potential, leading to prolonged repolarization, and extended cardiac intervals [7–10]. Ultimately, both respiratory arrest and cardiac toxicity can culminate in cardiac arrest.

Emergency medical services (EMS) personnel are on the front lines of the opioid crisis, playing a crucial role in responding to opioid overdoses [11]. If an overdose is suspected, current AHA guidelines recommend that naloxone administration be considered [5]. Naloxone is a fast-acting and competitive opioid antagonist that rapidly reverses the effects of an opioid overdose and restores spontaneous breathing within seconds to minutes [1,12]. There is strong evidence that naloxone plays a key role in the prevention of cardiac arrest [13,14].

Despite naloxone’s critical role as an antidote for opioid overdose, uncertainties persist regarding its use in opioid-associated cardiac arrest [5]. Research on the role of naloxone in the context of OA-OHCA remains limited. A recent ILCOR systematic review by Grunau et al. examined advanced life support interventions for OA-OHCA, including naloxone [15]. While their review addressed multiple therapies, our goal was to summarize patient-centred outcomes reported in studies of naloxone administration by EMS in suspected opioid-associated OHCA

## Methods

The protocol for this systematic review was registered in PROSPERO (CRD42024529257) and conducted in accordance with the Preferred Reporting Items for Systematic Reviews and Meta-Analyses (PRISMA) guidelines (Data in S1 File).

### Eligibility criteria

Studies were included if they reported naloxone administration by emergency medical services (EMS) for suspected opioid-associated out-of-hospital cardiac arrest and reported at least one patient-centred outcome. When studies explicitly identified OHCA cases attributed to opioid overdose, only those cases were included. In studies that did not report a discrete overdose-related OHCA subgroup, we included OHCA cases in which naloxone was administered by EMS, recognizing that naloxone exposure does not itself confirm overdose etiology.

There were no restrictions on the publication date or language. Animal studies and data from secondary sources such as case studies, opinion papers, reviews, meta-analyses, preprints, abstracts, and conference presentations were excluded. Conference abstracts and other grey literature identified during database searches were screened but were not included in the final analysis unless a corresponding full-text publication was available.

### Data sources and Search

In concert with an information specialist (CW), a search strategy (Data in S2 Appendix) was devised and underwent PRESS review [16]. A systematic search was conducted in OVID Medline, Embase, and Cochrane from database inception to December 2024. The bibliographies of selected papers were also searched for any missed articles.

### Study selection

All articles generated by the search were exported into the Covidence software (version 2.0, Veritas Health Innovation, Melbourne, Australia). This software was utilized for removing duplicates and screening processes. Screening occurred in three stages: (1) screening of titles, (2) screening of abstracts, (3) screening of full texts. Screening was conducted independently by two reviewers (HS and KB) based on predetermined inclusion criteria. Any disagreements between the study members were resolved through discussion with one of the senior authors (RM).

### Data extraction

Two reviewers (HS and KB) extracted data from included studies using a predefined data extraction form. The data extraction form included study information (study design, location, and objective) and quantitative data [ex. proportion, mean, median, odds ratio (OR), 95% confidence interval (95% CI)]. A single reviewer extracted data, followed by verification of the data by the second reviewer. Discrepancies were resolved by discussion.

### Outcomes

The primary outcome of interest was return of spontaneous circulation (ROSC). ROSC was selected as the primary outcome because it is consistently reported in studies including OHCA patients. Secondary outcomes included survival to hospital admission, survival to hospital discharge, neurological outcome at hospital discharge, survival up to one year, and adverse events.

Outcomes were extracted as reported in each study. Given expected heterogeneity in outcome definitions, naloxone administration and reporting across studies, outcomes were synthesiszed descriptively without attempts to standardize definitions or perform quantitative pooling.

### Risk of bias in individual studies

Two reviewers (HS and KB) independently assessed the risk of bias in included studies using the ROBINS-I tool (Risk of Bias In Non-randomized Studies – of Interventions), resolving conflicts through discussion [17]. The ROBINS-I tool evaluates the risk of bias in the results of non-randomized studies of interventions, comparing the health effects of two or more interventions. The certainty of evidence for each outcome was also rated using the GRADE (Grading of Recommendations, Assessment, Development and Evaluation) approach [18], considering study limitations, inconsistency, indirectness, imprecision, and publication bias.

### Data synthesis

A meta-analysis was considered but ultimately not performed due to the heterogeneity and observational nature of the included studies, which would likely not result in any meaningful pooling of results. Study findings are presented as a narrative synthesis.

## Results

### Study selection

**Fig 1:**
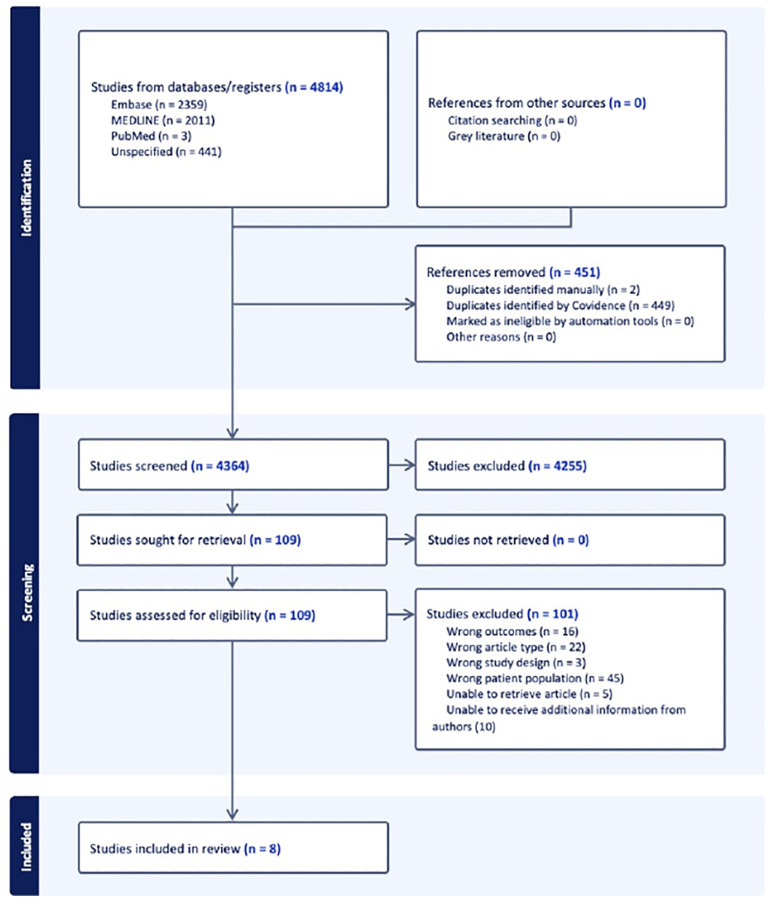
PRISMA flow diagram.

The literature search yielded a total of 4814 articles. After duplicates were removed, 4364 unique articles progressed to screening. 1604 titles were deemed irrelevant and were subsequently excluded, leaving 2760 abstracts for further consideration. After abstract screening, 109 articles were identified and made it to full-text screening. Following full-text assessment, eight studies met eligibility and were included in our review (Fig 1). In comparison, the ILCOR review included five studies related to naloxone in OA-OHCA, two of which were conference abstracts [15]. One of the full-text articles reported results from the same study as one of the conference abstracts by the same author.

We contacted the authors of 10 studies that might have met our eligibility criteria with selected data. However, these studies were ultimately excluded as we could not receive additional information on the outcomes of the population of interest. Discrepancies between reviewers occurred on three occasions and were resolved through discussion with one of the senior authors. Reviewer agreement during title and abstract screening was moderate (Kappa = 0.43) and was substantial (Kappa = 0.75) during full-text screening.

### Risk of bias

The ROBINS-I tool was used to assess whether studies had a low, moderate, serious, or critical risk of bias. Three of the studies were assessed to have an overall low risk of bias, four had a moderate risk of bias, and one study had a serious risk of bias (Table in S3 Table). The GRADE approach was utilized to assess the certainty of evidence. Outcomes including ROSC, survival to hospital admission, and survival to hospital discharge were rated as low certainty, while neurological outcome, adverse events and long-term survival were rated as very low certainty (Table in S4 Table).

### Study characteristics

Characteristics of the included studies are summarized in Table 1. Seven of the included studies were retrospective cohort studies conducted in the United States [19,20,22–26], and one was a prospective cohort study conducted in Denmark [21]. All eight studies reported outcomes for OHCA patients who received naloxone [19–26]. Four of the studies included comparators where outcomes were compared across more than one patient group. Koller at al. [20] compared OHCAs with a suspected overdose etiology with non-overdose OHCAs. Dillon et al. [19] and Quinn et al. [23] compared OHCAs who received naloxone with OHCA cases who did not receive naloxone. Strong et al. [26] compared OHCA cases given naloxone prior to vascular access with OHCA patients who did not receive naloxone prior to vascular access.

**Table 1:** Characteristics of included studies and drug-related OHCA patients.

Author, Year, Country, N	Study Design	Population	Data Collection Timeframe	Witness Status	Naloxone Administration (Route & Dose)	Other Drugs Given During Resuscitation	Initial ECG Rhythm
**Dillon et al.**, 2024, United States, 471	Retrospective cohort study	OHCA cohort; subset are drug related	2015–2023	Not reported	Not reported	Not reported	Not reported
**Koller et al.**, 2014, United States, 180	Retrospective cohort study	OHCA cohort; subset are drug related	2006–2008 Late 2009–2011	48.4% bystanders, 6.7% EMS	Not reported	Epinephrine, Sodium bicarbonate, atropine, amiodarone	VF/VT: 15.6%,
**Nielsen et al.**, 2011, Denmark, 352	Prospective cohort study	Overdose cohot; arrest subset	1994–2003	Not reported	IV: 0.8 mg SQ/IM: 0.4 mg	Not reported	Not reported
**Ornato et al.**,2023, United States, 22	Retrospective cohort study	Overdose cohot; arrest subset	Months before and after mid-March 2020	31.8% (7/22) (no further details)	IV, IN, IM, IO (Dosing not reported)	Not reported	VF/VT: 0%, PEA: 18.2% (4/22), Asystole: 45.5% (10/22)
**Quinn et al.**, 2024, United States, 175	Retrospective cohort study	OHCA cohort	2017–2022	Not reported	IN, IO, IV (Dosing not reported)	Epinephrine	VF/VT: 4.7% (8/172), PEA: 18% (31/172), Asystole: 77.3% (133/172)
**Saybolt et al.**, 2009, United States, 36	Retrospective cohort study	OA-OHCA	2003–2007	Not reported	19.0– 10 mg, IV	Epinephrine, atropine, dextrose, sodium bicarbonate, calcium chloride	VF/VT: 2.8% (1/36), PEA: 33.3% (12/36), Asystole: 63.9% (23/36)
**Sporer et al.**, 1996, Unites States, 16	Retrospective cohort study	Overdose cohort; arrest subset	1993	Not reported	88% received 2–4 mg; 13% received >4 mg, IM, IV, or ET	Not reported	Not reported
**Strong et al.**, 2024, United States, 42	Retrospective cohort study	OHCA cohort; subset are drug related	2018–2021	Not reported	Initial dose: 2 mg (IQR: 2–2), IM or IN	Epinephrine	100% non-shockable (PEA/asystole)

VF = Ventricular Fibrillation, VT = Ventricular Tachycardia, PEA = Pulseless Electrical Activity.

IV = intravenous, IN = intranasal, IM = intramuscular, IO = intraosseous SQ = subcutaneous, ET = endotracheal intubation.

The initial populations in the included studies varied (Table 1). OA-OHCA definitions and naloxone administration also differed. In several studies, investigators included all OHCA patients, of whom a subset were classified as drug-related based on EMS clinician impression using scene findings, witness reports, or patient history [19,23,26]. One study included only patients with OA-OHCA, defined by paramedic suspicion of opioid use at the scene [24]. Other studies included patients with suspected opioid overdose and reported outcomes for those found to be in cardiac arrest.

### Opioid-associated out-of-hospital cardiac arrest

Out-of-hospital cardiac arrests with overdose as a presumed etiology of arrest were the primary population of interest in the included studies. Three studies examined out-of-hospital overdose cases and reported outcomes for overdose patients found to be presenting in cardiac arrest [21,22,25]. Four studies looked at OHCA patients and reported separate outcomes for patients who received naloxone and were presumed to have an overdose etiology of arrest [19,20,23–26]. The remaining study only included patients who had a presumed opioid-associated OHCA [24].

### Resuscitation characteristics

Resuscitation characteristics such as witness status, naloxone dosing and route, medications other than naloxone, and the ECG rhythm of patients from the included studies are summarized in Table 1. The route and dose of naloxone administration were not reported in three of the studies. Intravenous and intramuscular methods of naloxone administration were reported in the other studies. Intraosseous, subcutaneous, and endotracheal tube routes of administration were also noted as routes of naloxone administration utilized. Medications other than naloxone that were given to patients included mainly epinephrine. However, other medications such as sodium bicarbonate, atropine, amiodarone, dextrose, and calcium chloride were also given during resuscitation in some of the studies.

Initial electrocardiogram (ECG) rhythm was reported in five of the studies. Ventricular fibrillation or ventricular tachycardia was a reported ECG rhythm in four studies and ranged from 0% – 15.6% [20,22–24]. The percentage of patients in pulseless electrical activity (PEA) and asystole was reported in three studies, and in these studies, PEA ranged from 18.2% – 33.3% while asystole ranged from 45.5% – 77.3 [22–24]. In the study by Strong et al. [24], 100% of the patients had initial non-shockable rhythms (PEA or asystole), consistent with their inclusion criteria.

### Resuscitation outcomes and survival

Return of spontaneous circulation (ROSC) was reported for patients in all eight studies (Table 2). The reported proportion of cardiac arrest patients who achieved ROSC ranged from 4.3%–50.0% [19–26]. Seven studies reported survival to hospital admission, which ranged from 11.1% – 55.0% [19,20,22,24–26]. Seven studies reported survival to discharge, and this ranged from 0% –20.4% [19,20,22–26]. Strong et al. [26] assessed neurologic status at discharge and found that 17% (7/42) of patients had a good neurologic outcome or a cerebral performance category score ≤ 2. Furthermore, there were no adverse events following naloxone administration reported in the publications of the included studies. Although data was sparse, no adverse events associated with intra-arrest naloxone were identified in the included studies. Furthermore, no studies reported on our pre-specified outcome of long-term survival (post-discharge from hospital).

**Table 2:** Outcomes for drug-related OHCA patients.

Author, Year	Number of patients	ROSC	Survival to hospital	Survival to discharge
Dillon et al., 2024	471	198/471 (42.0%)	259/471 (55.0%)	96/471 (20.4%)
Koller et al., 2014	180	25%*	34.2%*	18.9%*
Nielsen et al., 2011	352	15/352 (4.3%)	Not reported	Not reported
Ornato et al., 2023	22	6/22 (27.3%)	5/22 (22.7%)	1/22 (4.5%)
Quinn et al., 2024	175	59/175 (33.7%)	71/175 (40.6%)	8/175 (4.6%)
Saybolt et al., 2009	36	3/36 (8.3%)	4/36 (11.1%)	1/36 (2.8%)
Sporer et al., 1996	16	2/16 (12.5%)	2/16 (12.5%)	0/16 (0%)
Strong et al., 2024	42	21/42 *** (50.0%)	21/42*** (50.0%)	8/42*** (19.0%)
**Totals:**	**1294**	349/1294(27%) **	424/942 (45%) **	148/942 (16%) **

* Raw numbers for the outcome were not available.

** Total includes raw counts from studies. For the study by Koller et al., which reports only percentages, estimated counts were included to calculate the total.

*** Reported outcomes represent patients with presumed drug-related OHCA who received naloxone prior to vascular access: outcomes for the overall drug-related OHCA cohort differed in the original study.

Quinn et al. [23] compared OHCA patients who were given naloxone to OHCA who were not given naloxone. They found no significant difference in survival to hospital discharge (p-value = 0.081), or ROSC (p-value = 0.779). Similarly, naloxone administration was not associated with greater odds of ROSC (OR, 0.43; 95% CI, 0.16–1.2; p-value, 0.79) or survival to discharge (OR, 1.01; 95% CI, 0.46–2.21; p-value, 0.99). In comparison, Dillon et al. [19] found that drug-related OHCAs treated with naloxone had greater survival to hospital discharge than presumed drug-related OHCAs not treated with naloxone (OR, 2.48; 95% CI, 1.34–4.58 vs OR, 0.91; 95% CI, 0.54–1.53), as well as greater ROSC (OR, 2.45; 95% CI, 1.56–3.83 vs OR, 0.81; 95% CI, 0.55–1.18).

Strong et al. [26] examined the role of early naloxone administration and found that OHCA patients with an initial non-shockable rhythm who received naloxone prior to vascular access had better outcomes, including ROSC at any time (aOR, 2.14; 95% CI, 1.20–3.81), ROSC at ED arrival (aOR, 2.14; 95% CI, 1.18–3.88), survival to admission (aOR, 2.86; 95% CI, 1.60–5.09), survival to discharge (aOR, 4.41; 95% CI, 1.78–10.97), and good neurologic outcome (aOR, 4.61; 95% CI, 1.74–12.19). OHCA patients with a presumed overdose etiology made up most of the group that received naloxone before vascular access.

## Discussion

This systematic review examined outcomes associated with EMS-administered naloxone for opioid-associated out-of-hospital cardiac arrest (OA-OHCA). 4814 studies were screened; eight met eligibility and were included. A recent ILCOR systematic review by Grunau et al. evaluated opioid-specific advanced life support interventions during OA-OHCA using studies with explicit comparator groups and found very low-certainty evidence with no convincing benefit [15]. In contrast, the present review adopted broader inclusion criteria to summarize outcomes reported in naloxone-exposed overdose-related OHCA cohorts, including studies without formal comparators. This broader inclusion criterion led to the inclusion of an additional 5 full-text manuscripts in our study. Our review does not provide additional comparative evidence regarding naloxone effectiveness, but instead highlights the heterogeneity in naloxone timing and overdose ascertainment across the available literature. Additionally, although both our review and the ILCOR review used the ROBINS-I tool, the risk-of-bias judgments differed for overlapping studies. This might have been because the ILCOR review evaluated them within a comparative effectiveness framework requiring explicit comparator groups, whereas our review included exposure-defined cohorts and synthesized outcomes descriptively. Our overall conclusions are consistent with those of the ILCOR review.

Results from the included studies revealed that some OA-OHCA patients had clinical improvements following resuscitation and naloxone administration, but survival was ultimately low. For patients who survived to hospital admission, few survived to hospital discharge. This finding is not surprising as OHCAs in general are complex and associated with poor prognosis [27]. Overdoses involving multiple drugs are becoming more common, with an increase observed in the incidence of opioid stimulant OHCAs [28]. The drugs implicated in the arrests of the patients from the included studies cannot be ascertained. It is possible that some of these patients suffered from a polypharmacy overdose which would further add to the complexity as naloxone may not improve outcomes for these patients.

Among cardiac arrest outcomes, ROSC was extremely variable across studies. Strong et al. [26] reported the highest ROSC of 50%, despite only including patients who presented with an initial non-shockable rhythm. Similarly, Quinn et al. [23] reported a ROSC of 33.7% despite 95% of patients presenting with a non-shockable rhythm. This is interesting as studies have found that shockable rhythms are associated with better outcomes including ROSC and survival for OHCA patients [29,30]. Other studies examining drug related OHCAs similarly reported a lower percentage of patients presenting with a shockable rhythm. Yogeswaran et al. [28] and Orkin et al. [31] both reported that 8% of their drug related OHCAs presented with a shockable rhythm. Paredes et al. [32] found the percentage of overdose related OHCAs presenting in VF/VT to be 6%. In comparison, the included study by Koller et al. [20] reported a substantially higher percentage of patients (15.6%) presenting with a shockable rhythm (VF/VT) but a lower ROSC percentage of 25%. It is possible that the patient population in the study by Koller et al. was not composed solely of opioid-related overdoses and so naloxone did not improve outcomes despite a higher percentage of patients presenting in VF/VT.

The percentage of patients with ROSC was higher in the newer studies, with the three studies published in 2024 reporting the highest percentages of ROSC of 50%, 42%, and 33.7%. It could be that the patient population in these studies were more responsive to resuscitation efforts, or that there is a temporal improvement in clinical outcomes associated with drug related OHCAs in recent years. The latter hypothesis would be consistent with studies that have found improvements in outcomes associated with OHCA over time [33–35], however, further investigation would be required to determine if this is the case. It is also possible that a subset of patients in these studies were in ‘pseudo-PEA’ where their pulse may not have been readily detectable, yet they still retained cardiac activity, allowing for great potential to benefit from naloxone [26,36].

Current resuscitation guidelines from the International Liaison Committee on Resuscitation (ILCOR) and American Heart Association (AHA) note that naloxone administration may be reasonable for adults in OA-OHCA if administration does not distract from other life-saving interventions, including compressions and ventilations [37]. However, the available evidence remains limited, heterogeneous, and largely observational, and the role of naloxone during OHCA remains uncertain.

The timing of naloxone administration may be a crucial factor which impacts the efficacy of the resuscitation of OA-OHCA. Strong et al. [26] examined the role of early naloxone administration and found that OHCA patients with an initial non-shockable rhythm who received naloxone early or before vascular access had better clinical outcomes, including ROSC, survival, and good neurologic outcome. OHCA patients with a presumed overdose etiology made up most of the group that received naloxone before vascular access. These findings, and the well-known time dependent effects of other standard advanced cardiac life support (ACLS) medications [38,39], may speak to the potential benefit that early recognition may have in improving outcomes for OA-OHCA patients.

This systematic review had several limitations that should be noted. First, many of the included studies did not have a comparator/control group, and there was substantial heterogeneity in how OA-OHCA was defined in the included studies, so the true benefit (if any) or risks of naloxone cannot be ascertained. Some studies defined drug-related arrest using naloxone administration as a proxy, others relied on EMS clinician impression, and others evaluated opioid overdose cohorts with a cardiac arrest subset. Second, EMS may have been more likely to presume an overdose etiology for arrest in patients who responded to naloxone, and this would introduce bias, as OA-OHCA patients who did not respond to naloxone may not have been categorized as such in the included studies. Third, due to a lack of information on the timing of naloxone administration, as well as a wide variation in the reported naloxone dosages and route of administration, we could not draw conclusions on best practices for naloxone administration. Naloxone was not uniformly administered during active cardiac arrest across studies. Importantly, Dillon et al. demonstrate that naloxone is frequently administered during OHCA even when opioid overdose is not ultimately documented as the presumed etiology of arrest, meaning that naloxone exposure alone may not reliably define opioid-associated OHCA. Fourth, it is unknown if opioids were the only drug implicated in the arrests or whether patients suffered an overdose involving multiple drugs, however, the latter is more likely. Lastly, the total sample size for OA-OHCA patients was 1294 from all the included studies. This sample size limits our ability to draw meaningful conclusions on the reported outcomes and limits the generalizability. Larger-scale prospective or retrospective studies of OA-OHCA patients with a comparator may be helpful in determining the role of naloxone in these patients. Furthermore, more detailed reporting of naloxone administration, including dosing, route, and timing, as well as clear definitions of OA-OHCA, and inclusion of appropriate comparator groups, is needed in future studies to determine whether naloxone administration during OHCA is beneficial.

## Conclusion

There are a limited number of retrospective and prospective studies assessing the use of naloxone in patients who present in opioid-associated out-of-hospital cardiac arrest (OA-OHCA). Furthermore, the lack of randomized controlled trials and the substantial heterogeneity in case definitions and naloxone timing in the current literature make it impossible to draw causal inferences of naloxone effectiveness during cardiac arrest. Our findings are consistent with the ILCOR review, emphasizing the need for higher-quality research evaluating naloxone during OA-OHCA. Future research should prioritize standardizing the definition of OA-OHCA, including appropriate comparator groups, and provide detailed reporting of naloxone administration, including dose, route, and timing.

## Supporting information

S1 File PRISMA checklist. (PDF)

S2 Appendix Search strategy. (DOCX)

S3 Table Risk of bias assessment for included studies. (DOCX)

S4 Table Summary of findings (GRADE). (DOCX)
