# Supplementary material for: Use of naloxone by EMS for opioid-associated out-of-hospital cardiac arrest and associated patient-centered outcomes: A systematic review
Source: PLoS One. 2026 Jun 17;21(6):e0351738. doi: 10.1371/journal.pone.0351738 (PMC13274882; doi:10.1371/journal.pone.0351738)
Supplement: S2 Appendix — (DOCX) [file pone.0351738.s002.docx]

MEDLINE Search Strategy

Database: Ovid MEDLINE(R) ALL Search Strategy:

--------------------------------------------------------------------------------

1 naloxone/ or buprenorphine, naloxone drug combination/ or naltrexone/

2 (naloxone or narcan or naltrexone).ti,ab,kf,kw.

3 heart arrest/ or out-of-hospital cardiac arrest/

4 ((heart or cardiac or cardiopulmonary or respiratory) adj2 arrest).ti,ab,kf,kw.

5 Opiate Overdose/

6 exp Opioid-Related Disorders/

7 exp Analgesics, Opioid/

8 ((opiate* or opioid*) adj3 (abus* or misus* or addict* or disorder* or overdose* or depend*)).ti,ab,kf,kw.

9 ((opioid* or opiate*) adj3 (induc* or associat*)).ti,ab,kf,kw.

10 (opioid* or opiate*).ti,ab.

11 emergency medical services/ or ambulances/ or air ambulances/

12 paramedics/

13 Ambulances/

14 EMS.ti.

15 (paramedic* or ambulance*).ti,ab,kf,kw.

16 ("first responder*" or rescue*).ti,ab,kf,kw.

17 firefight*.ti,ab,kf,kw.

18 (emergenc* adj2 (responder* or medic* or worker* or transport* or dispatch* or personnel or

technician*)).ti,ab,kf,kw.

19 (prehospital or "out of hospital" or "out-of-hospital").ti,ab,kf,kw.

20 (emergenc* adj1 (medical or health) adj1 (service* or care)).ti,ab.

21 Respiratory Insufficiency/

22 (bystand* or samaritan or layperson*).ti,ab,kw,kf.

23 EMT.ti.

24 police*.ti,ab,kw,kf.

25 (alfentanil or alphaprodine or buprenorphine or butorphanol or codeine or dextromoramide or dextropropoxyphene or

dihydromorphine or diphenoxylate or ethylketocyclazocine or ethylmorphine or etorphine or fentanyl or heroin or hydrocodone or hydromorphone or levorphanol or meperidine or meptazinol or methadone or methadyl acetate or morphine or nalbuphine or opium or oxycodone or oxymorphone or pentazocine or phenazocine or phenoperidine or pirinitramide or promedol or remifentanil or sufentanil or tapentadol or tilidine or tramadol).ti,ab,kf,kw.

26 ((respiratory or ventilatory) adj2 (failure* or depression* or insufficiency)).ti,ab,kw,kf.

27 1 or 2

28 3 or 4 or 21 or 26

29 5 or 6 or 7 or 8 or 9 or 10 or 25

30 11 or 12 or 13 or 14 or 15 or 16 or 17 or 18 or 19 or 20 or 22 or 23 or 24

31 27 and 28 and 29 and 30 [*focus* results]

32 27 and 28 and 29

33 27 and 28 and 30

34 27 and 29 and 30

35 32 or 33 or 34

36 35 not 31 [*broad* results]

**EMBASE Search Strategy**

| Search Statement |
| --- |
| 1 exp naloxone/ or exp buprenorphine plus naloxone/ |
| 2 exp naltrexone/ |
| 3 (naloxone or narcan or naltrexone).ti,ab,kf,kw. |
| 4 exp heart arrest/ |
| 5 exp "out of hospital cardiac arrest"/ |
| 6 exp respiratory failure/ |
| 7 ((heart or cardiac or cardiopulmonary or respiratory) adj2 arrest).ti,ab,kf,kw. |
| 8 ((respiratory or ventilatory) adj2 (failure* or depression* or insufficiency)).ti,ab,kw,kf. |
| 9 exp opiate overdose/ |
| 10 exp opiate addiction/ |
| 11 exp opiate/ |
| 12 ((opiate* or opioid*) adj3 (abus* or misus* or addict* or disorder* or overdose* or depend*)).ti,ab,kf,kw. |
| 13 ((opioid* or opiate*) adj3 (induc* or associat*)).ti,ab,kf,kw. |
| 14 (opioid* or opiate*).ti,ab. |
| 15 (alfentanil or alphaprodine or buprenorphine or butorphanol or codeine or dextromoramide or dextropropoxyphene or dihydromorphine or diphenoxylate or ethylketocyclazocine or ethylmorphine or etorphine or fentanyl or heroin or hydrocodone or hydromorphone or levorphanol or meperidine or meptazinol or methadone or methadyl acetate or morphine or nalbuphine or opium or oxycodone or oxymorphone or pentazocine or phenazocine or phenoperidine or pirinitramide or promedol or remifentanil or sufentanil or tapentadol or tilidine or tramadol).ti,ab,kf,kw. |
| 16 exp emergency health service/ |
| 17 exp ambulance/ |
| 18 exp air medical transport/ |
| 19 exp paramedical personnel/ |
| 20 EMS.ti. |
| 21 (paramedic* or ambulance*).ti,ab,kf,kw. |
| 22 ("first responder*" or rescue*).ti,ab,kf,kw. |
| 23 firefight*.ti,ab,kf,kw. |
| 24 (emergenc* adj2 (responder* or medic* or worker* or transport* or dispatch* or personnel or technician*)).ti,ab,kf,kw. |
| 25 (prehospital or "out of hospital" or "out-of-hospital").ti,ab,kf,kw. |
| 26 (emergenc* adj1 (medical or health) adj1 (service* or care)).ti,ab. |
| 27 (bystand* or samaritan or layperson*).ti,ab,kw,kf. |
| 28 EMT.ti. |
| 29 police*.ti,ab,kw,kf. |
| 1 or 2 or 3 |
| 4 or 5 or 6 or 7 or 8 |
| 9 or 10 or 11 or 12 or 13 or 14 or 15 |
| 16 or 17 or 18 or 19 or 20 or 21 or 22 or 23 or 24 or 25 or 26 or 27 or 28 or 29 |
| 30 and 31 and 32 and 33 |
| 30 and 31 and 32 |
| 30 and 31 and 33 |
| 30 and 32 and 33 |
| 35 or 36 or 37 |
| 38 not 34 |
| 34 or 35 or 36 or 37 |
| limit 40 to "remove medline records" |
| limit 34 to "remove medline records" |
| 41 not 42 |
