# Supplementary material for: Use of naloxone by EMS for opioid-associated out-of-hospital cardiac arrest and associated patient-centered outcomes: A systematic review
Source: PLoS One. 2026 Jun 17;21(6):e0351738. doi: 10.1371/journal.pone.0351738 (PMC13274882; doi:10.1371/journal.pone.0351738)
Supplement: S3 Table — (DOCX) [file pone.0351738.s003.docx]

| **S3 Table: Risk of Bias Assessment for Included Studies** | | | | | | | | |
| --- | --- | --- | --- | --- | --- | --- | --- | --- |
| **Study** | **Confounding** | **Selection** | **Measurement**  **of**  **Intervention** | **Deviation from Intervention** | **Missing Data** | **Measurement of**  **Outcomes** | **Reported Result** | **Risk of**  **Bias** |
| Dillon 2024 | Moderate | Low | Moderate | Low | Moderate | Low | Low | Low |
| Koller  2014 | Serious | Low | Low | Low | Low | Low | Low | Moderate |
| Nielsen 2011 | Serious | Low | Low | Low | Moderate | Low | Low | Moderate |
| Ornato 2023 | Serious | Low | Low | Low | Low | Low | Low | Moderate |
| Quinn  2024 | Low | Low | Low | Low | Low | Low | Low | Low |
| Saybolt 2009 | Serious | Low | Low | Serious | Serious | Low | Low | Serious |
| Sporer  1996 | Serious | Moderate | Low | Low | Low | Low | Low | Moderate |
| Strong 2024 | Serious | Moderate | Low | Low | Low | Low | Low | Low |
