# Supplementary material for: Use of naloxone by EMS for opioid-associated out-of-hospital cardiac arrest and associated patient-centered outcomes: A systematic review
Source: PLoS One. 2026 Jun 17;21(6):e0351738. doi: 10.1371/journal.pone.0351738 (PMC13274882; doi:10.1371/journal.pone.0351738)
Supplement: S4 Table — (DOCX) [file pone.0351738.s004.docx]

| **S4 Table: Summary of Findings (GRADE)** | | | | |
| --- | --- | --- | --- | --- |
| **Outcome** | **Number of Studies** | **Number of Participants** | **Certainty (GRADE)** | **Rationale** |
| ROSC | 8 | 349 | Low | Observational studies,  Inconsistency: Difference in ROSC rates across studies |
| Survival to Hospital Admission | 6 | 424 | Low | Observational studies,  Inconsistency: Difference in survival rates across studies |
| Survival to Hospital Discharge | 7 | 148 | Low | Observational studies,  Inconsistency: Difference in survival rates across studies |
| Neurological Outcome | 1 | 7 | Very Low | Single study, small sample, non-randomized study |
| Adverse Events | 0 | 0 | Very Low | No studies reported; evidence absent for this pre-specified outcome. |
| Long-Term Survival  (post-discharge) | 0 | 0 | Very Low | No studies reported; evidence absent for this pre-specified outcome. |
